# Biochemical Properties of Human D-Amino Acid Oxidase

**DOI:** 10.3389/fmolb.2017.00088

**Published:** 2017-12-15

**Authors:** Giulia Murtas, Silvia Sacchi, Mattia Valentino, Loredano Pollegioni

**Affiliations:** ^1^Dipartimento di Biotecnologie e Scienze della Vita, Università degli Studi dell'Insubria, Varese, Italy; ^2^The Protein Factory, Politecnico di Milano and Università degli Studi dell'Insubria, Milan, Italy; ^3^Sezione Adolfo Quilico, Istituto di Chimica del Riconoscimento Molecolare, CNR, Milan, Italy

**Keywords:** D-serine, D-amino acid oxidase, D-cysteine, substrate specificity, structure-function relationships

## Abstract

D-amino acid oxidase catalyzes the oxidative deamination of D-amino acids. In the brain, the NMDA receptor coagonist D-serine has been proposed as its physiological substrate. In order to shed light on the mechanisms regulating D-serine concentration at the cellular level, we biochemically characterized human DAAO (hDAAO) in greater depth. In addition to clarify the physical-chemical properties of the enzyme, we demonstrated that divalent ions and nucleotides do not affect flavoenzyme function. Moreover, the definition of hDAAO substrate specificity demonstrated that D-cysteine is the best substrate, which made it possible to propose it as a putative physiological substrate in selected tissues. Indeed, the flavoenzyme shows a preference for hydrophobic amino acids, some of which are molecules relevant in neurotransmission, i.e., D-kynurenine, D-DOPA, and D-tryptophan. hDAAO shows a very low affinity for the flavin cofactor. The apoprotein form exists in solution in equilibrium between two alternative conformations: the one at higher affinity for FAD is favored in the presence of an active site ligand. This may represent a mechanism to finely modulate hDAAO activity by substrate/inhibitor presence. Taken together, the peculiar properties of hDAAO seem to have evolved in order to use this flavoenzyme in different tissues to meet different physiological needs related to D-amino acids.

## Introduction

**Scheme 1:**
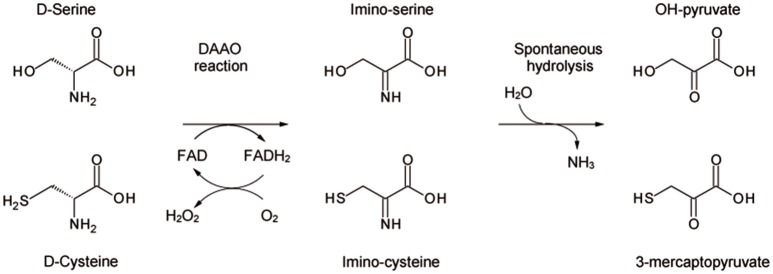
Scheme of the reaction catalyzed by DAAO on the physiological substrates D-serine and D-cysteine.

D-amino acid oxidase (DAAO, EC 1.4.3.3) is the FAD-containing enzyme that catalyzes the strictly stereospecific oxidative deamination of the D-isomer of neutral amino acids. Since its discovery in pig kidney (Krebs, 1935), DAAO has been widely studied as a prototype of FAD-dependent oxidases (Mattevi et al., 1996; Pilone, 2000). D-Amino acids are oxidized by DAAO to give the corresponding imino acids (subsequently non-enzymatically hydrolyzed to α-keto acids and ammonia) and hydrogen peroxide (which is generated during the reoxidation of FADH_2_ on molecular oxygen (Scheme 1).

DAAO plays various roles in different organisms: in microorganisms it catalyzes the catabolism of D-amino acids for cell metabolism, and specific functions have been proposed in nematodes, insects, and lower vertebrates (Pollegioni et al., 2007; Saitoh et al., 2012). In higher vertebrates, it was thought to have a detoxifying function in catabolizing exogenous D-amino acids. In the last few years, however, a key role in the metabolism of D-amino acids with specific physiological function has come into play. In mammals, DAAO is mainly present in kidney, liver, and brain. In the latter, D-serine is the most abundant D-amino acid (Weatherly et al., 2017). D-Serine binds to N-methyl-D-aspartate-type glutamate receptor (NMDAR) - specifically at the “strychnine-insensitive glycine modulatory site” (Mothet et al., 2000). In the brain, NMDARs are involved in main physiological processes such as development, neuronal cell migration, plasticity, learning and memory (Wolosker et al., 1999, 2008; Mothet et al., 2000; Snyder and Kim, 2000; Foltyn et al., 2005; Martineau et al., 2006). In the central nervous system (CNS) *DAAO* gene and protein expression show an opposite content and distribution when compared with D-serine level (Horiike et al., 1994). The physiological activity of NMDAR is modulated by D-serine cellular concentration, which level is partially controlled by DAAO-induced catabolism (Sacchi et al., 2012).

An excessive production / release of D-serine has been related to acute and chronic degenerative disorders (such as stroke, amyotrophic lateral sclerosis, epilepsy, and Parkinson's, Alzheimer's, and Huntington's diseases); an abnormal decrease in D-serine concentration was instead reported in psychiatric disorders (i.e., schizophrenia and bipolar disorders) (Rossi et al., 2007; Labrie and Roder, 2010; Mustafa et al., 2010; Fuchs et al., 2011; Lu et al., 2011). Here, the R199W mutation in DAAO, yielding a loss of enzymatic activity, has been associated with a familial case of amyotrophic lateral sclerosis (Mitchell et al., 2010) and association studies have linked DAAO (as well as its main modulatory partner pLG72) with schizophrenia susceptibility; (for a review, see Sacchi et al., 2012, 2016).

DAAO is mainly a peroxisomal enzyme (Horiike et al., 1994; Moreno et al., 1999) although an active DAAO form has been identified in the cytosol, both in glial cells (Sacchi et al., 2008, 2011) and neurons (Popiolek et al., 2011). Notably, DAAO was recently reported to be present in cytosol and nuclei of rat proximal tubule epithelial cells treated with propiverine (Luks et al., 2017), too, and to be secreted into the lumen by intestinal epithelial cells (Sasabe et al., 2016).

In past decades, extensive investigations were performed on pig kidney and yeast DAAOs (Pollegioni et al., 1993, 2007). Later on, recombinant human DAAO (hDAAO) expressed in *E. coli* was purified as active holoenzyme (347 residues), containing one molecule of non-covalently bound FAD per 40 kDa-protein monomer. hDAAO shows the spectral properties of the FAD-containing flavoenzymes (Molla et al., 2006). From a structural point of view, DAAO tertiary structure is made of two domains, each generated by several non-contiguous sequence regions: an interface domain made of a large, twisted, antiparallel β-sheet and a FAD-binding domain possessing the dinucleotide binding motif (Kawazoe et al., 2006).

hDAAO possesses a number of distinctive properties: (a) the weakest interaction with the flavin cofactor among known DAAOs (K_d_ = 8 × 10^−6^ M). In the presence of an active site ligand (such as benzoate) a 20-fold tighter interaction with FAD is apparent (Molla et al., 2006). The flavin molecule shows an elongated conformation and it is completely buried in the protein core with the only exception of the isoalloxazine ring, which is accessible to the solvent. The conformation of a short hydrophobic stretch (^47^VAAGL^51^) near the *si*-face of the isoalloxazine ring is significantly different between human and porcine DAAO and thus was considered responsible for the comparatively lower FAD affinity of hDAAO (Kawazoe et al., 2006); (b) it exists in solution as an equilibrium between the active holoenzyme and the inactive apoprotein; (c) it is a stable 80-kDa homodimer in both the holo- and the apoprotein form (Molla et al., 2006), whereas all known DAAO apoproteins are monomeric. In the homodimer, the two subunits show a head-to-head mode of interaction, as also observed in other mammalian DAAOs (Mattevi et al., 1996; Pollegioni et al., 2007; Frattini et al., 2011); however, the dimerization interface is mostly positively charged in the enzyme from pig kidney (Mattevi et al., 1996) while in hDAAO it is mainly negatively charged (Kawazoe et al., 2006); d) its half-life (*in vitro* as well as at the cellular level) and activity are negatively affected by pLG72 binding (Sacchi et al., 2008, 2011; Cappelletti et al., 2014).

The hDAAO reaction follows a sequential kinetic mechanism. The product release from the reoxidized enzyme represents the rate-limiting step, as also observed for pig DAAO; the rate of flavin reduction is slower for the human enzyme (Molla et al., 2006). Small, uncharged D-amino acids seem the preferred substrates for hDAAO, showing a low substrate affinity and catalytic efficiency on D-serine, the physiological substrate in the brain. The volume of hDAAO active site is ~220 A^3^. Tyr224 is part of the active site loop: it has been proposed that it switches during substrate binding from an open to a closed conformation and thus to allow the binding of substrates possessing large side chains (Molla et al., 2006; Kawazoe et al., 2007a,b). Indeed, the switch of this loop, which improves the efficiency of the hydride transfer reaction by increasing the hydrophobicity of the active site, represents the rate-limiting step in the kinetic cycle.

Owing to the important physiological and pathological role of hDAAO, we decided to deepen the investigation on some of the key biochemical properties of the recombinant enzyme. The novel information on hDAAO has been discussed with regard to known properties, the final aim being to highlight its peculiar characteristics.

## Materials and methods

### Enzyme preparation

Recombinant hDAAO was expressed in *E. coli* cells and purified as reported in Molla et al. (2006) and Romano et al. (2009)*;* 40 μM of free FAD was present during all purification steps. The final enzyme preparation was stored in 20 mM Tris-HCl buffer, pH 8.0, 100 mM NaCl, 10% (v/v) glycerol, 5 mM 2-mercaptoethanol, and 40 μM FAD. hDAAO apoprotein was prepared by overnight dialysis of the holoenzyme against 1 M KBr (Molla et al., 2006). hDAAO concentration was determined based on the extinction coefficient at 445 nm for the holoenzyme (12.2 mM^−1^ cm^−1^) and at 280 nm for the apoprotein (75.2 mM^−1^ cm^−1^).

### Activity assay and kinetic measurements

DAAO activity was assayed with an oxygen electrode at air saturation, pH 8.5 and 25°C, using 28 mM D-alanine as substrate in the presence of 0.2 mM FAD (Molla et al., 2006). The apparent kinetic parameters were calculated according to a Michaelis–Menten equation using the initial velocity values determined at increasing substrate concentrations, i.e. up to 450 mM D-cycloserine, 90 mM D-cysteine, 90 mM D,L-DOPA and 27.5 mM D-kynurenine. The effect of MgCl_2_ and CaCl_2_ (1 or 10 mM), of 10 μM ATP or GTP and of both divalent ions and nucleotides was assessed using the standard activity assay.

The same assay was employed to investigate the pH and the temperature effects on the enzyme activity and stability. For pH-stability, residual activity was assayed after 30 or 60 min of incubation at different pH-values in a multicomponent buffer (15 mM Tris-HCl, 15 mM Na_2_CO_3_, 15 mM H_3_PO_4_, 100 mM KCl, and 1% (v/v) glycerol) (Harris et al., 1999); for the effect of temperature, the incubation was carried out in 10 mM potassium phosphate buffer, pH 8.0, containing 1% (v/v) glycerol, 5 mM 2-mercaptoethanol and 40 μM FAD. The effect of temperature on the rate constant of loss of enzymatic activity was fit based on the Arrhenius equation. To investigate the pH and the temperature effect on enzyme activity, hDAAO activity was assayed at: (a) 25°C, using 200 mM D-alanine in multicomponent buffer (Harris et al., 2001) containing 40 μM FAD at different pH-values; (b) different temperatures using 28 mM D-alanine in 75 mM sodium pyrophosphate, pH 8.5, containing 200 μM FAD. Experimental data for pH dependence were fit based on the equation for two dissociations (*Y* = *a* + *b*(10^pH–*pKa*1^)/(1 + 10^pH–*pKa*1^) + [*b* – *c*(10^pH–*pKa*2^)/(1 + 10^pH–*pKa*2^)]) or three dissociations (*Y* = *a* + *b*(10^pH–*pKa*1^)/(1 + 10^pH–*pKa*1^) + [*b* – *c*(10^pH–*pKa*2^)/(1 + 10^pH–*pKa*2^) + [*c* – *d*(10^pH–*pKa*3^)/(1 + 10^pH–*pKa*3^)]) (Harris et al., 2001).

### Spectral measurements

Circular dichroism (CD) spectra were recorded using a Jasco J-815 spectropolarimeter (Jasco Co., Cremella, Italy) in 10 mM Tris-HCl, pH 8.0, 1% (v/v) glycerol, and 40 μM FAD. The cell path length was 0.1 cm for measurements in the 200- to 250-nm region (0.1 mg protein/mL) and 1 cm for measurements in the 250–350 nm range (0.4 mg protein/mL) (Caldinelli et al., 2010).

Protein fluorescence spectra were measured at 1 μM protein concentration (0.04 mg/mL) in 20 mM Tris-HCl buffer, pH 8.0, 100 mM NaCl, 10% (v/v) glycerol, 5 mM 2-mercaptoethanol, and 40 μM FAD for the holoenzyme and in 50 mM sodium pyrophosphate, pH 8.3 for the apoprotein; spectra were recorded using a Jasco FP-750 instrument and were corrected for the buffer contribution. Protein fluorescence spectra were recorded between 300 and 400 nm, with excitation at 280 nm. The ligand dissociation constants (K_d_) were estimated by titrating 1 μM enzyme with increasing amounts of FAD, benzoate, or CBIO and following the protein fluorescence quenching at 330–340 nm (Molla et al., 2006). The K_d_-values for FAD to the hDAAO apoprotein moiety was determined in free form and in complex with benzoate (7 or 70 μM). In all cases, K_d_-values were determined using an hyperbolic plot.

## Results

### Effect of pH and temperature on hDAAO activity and stability

**Figure 1:**
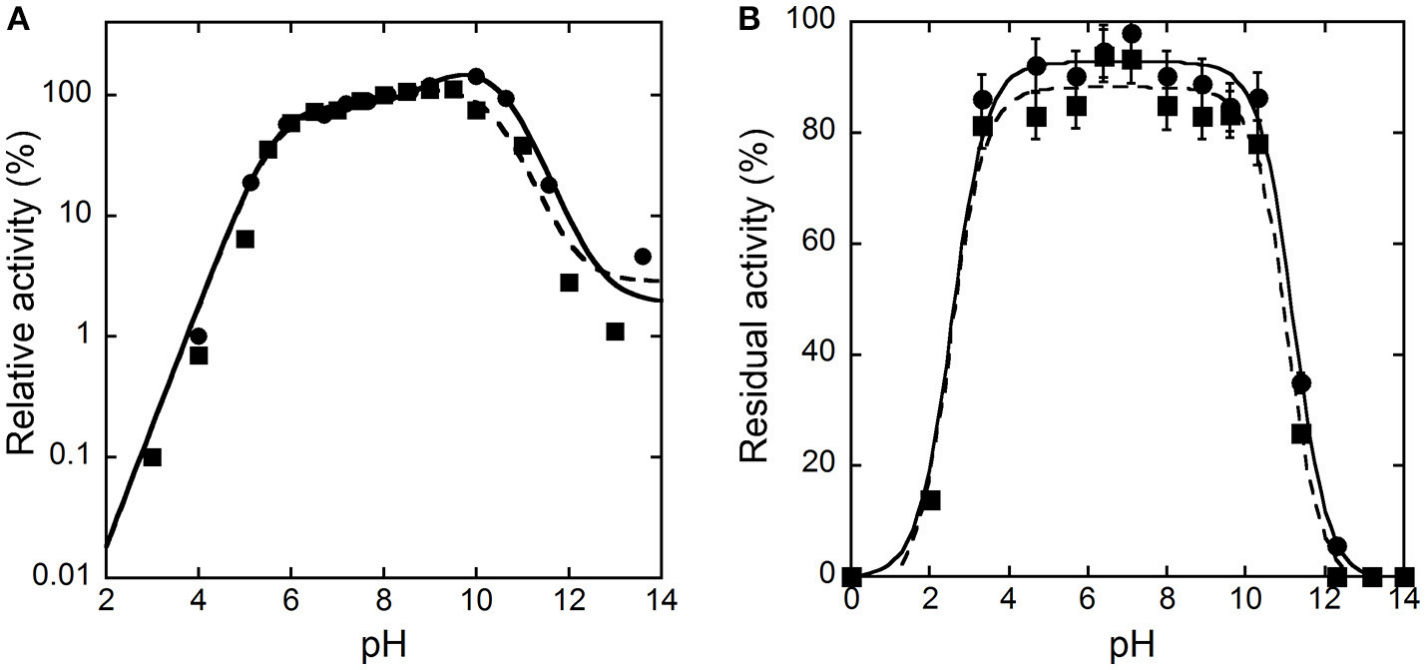
Effect of pH on the activity **(A)** and stability **(B)** of hDAAO. **(A)** The activity was assayed on D-Ala (●, **——**) or D-Ser (■, ---), at 25°C and air saturation; the value at pH 8.0 was reported as 100%. Data points were interpolated based on the equation for three dissociations (pK_a_s of 5.7, 9.6, 10.2 and of 5.6, 7.9, 10.5 for D-alanine and D-serine, respectively). **(B)** The residual activity of hDAAO was assessed after 30 (●, **——**) or 60 (■, ---) min of incubation at 4°C at the indicated pH values, and is related to the value determined at time = 0 min for each condition. Data points were fitted based on the equation for two dissociations. Each value was mean ± SD, *n* = 3; when not shown error bars are smaller than the symbol used.

The activity of hDAAO on D-alanine and D-serine increases with pH, reaching a maximal value at pH 10 and 9.5, respectively, and decreases quickly at higher values (Figure 1A). Concerning the pH effect on stability, the activity values determined after 30 or 60 min show that hDAAO is fully stable in a pH range of 4–10 (Figure 1B); experimental values were fitted based on the equation for two dissociations: estimated p*K*_*a*_'*s* were 2.5 ± 0.1 and 11.1 ± 0.1.

**Figure 2:**
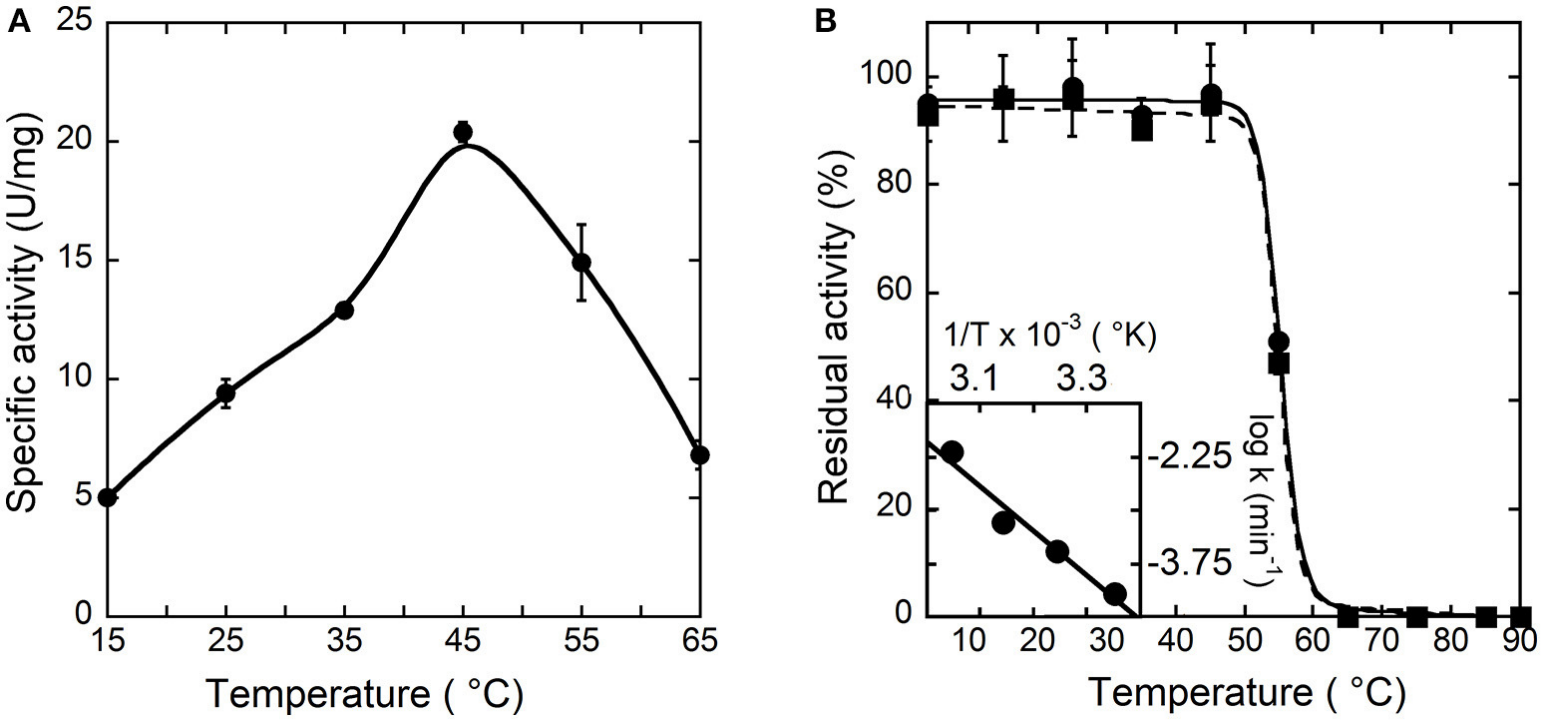
Effect of temperature on the activity **(A)** and stability **(B)** of hDAAO. **(A)** Activity values are reported as specific activity determined at a saturating D-alanine concentration (28 mM). The specific activity at 25°C (9.5 U/mg protein) corresponds to a k_cat_-value of 6 s^−1^. **(B)** The residual activity of hDAAO was assessed after 30 (●, **——**) and 60 (■, ---) min of incubation at pH 8.0 in 10 mM potassium phosphate buffer, 1% (v/v) glycerol, 5 mM 2-mercaptoethanol and 40 μM FAD, and is related to the value determined at time = 0 min for each condition. The rate constant for the enzyme inactivation at different temperatures were calculated and used for the Arrhenius plot reported as inset of **(B)**. Each value was mean ± SD, *n* = 3.

To investigate the temperature dependence of the initial rate of the reaction catalyzed by hDAAO, the enzymatic activity was assayed at different temperature values on 28 mM D-alanine (a saturating substrate concentration), at pH 8.5, and in the presence of 200 μM FAD by measuring the O_2_ consumption. As shown in Figure 2A, the optimal temperature of hDAAO activity is 45°C. The effect of temperature on the enzyme stability was evaluated assaying the residual activity after 30 or 60 min of incubation. The flavoenzyme was fully stable after 60 min of incubation at 45°C and fully inactivated at ≥65°C (Figure 2B). Melting temperatures were 55.2 and 54.9°C after 30 and 60 min of incubation, respectively, higher than T_m_'s determined from changes in protein fluorescence (Caldinelli et al., 2010). This indicates that loss of enzymatic activity follows the alteration in secondary and tertiary structure, a behavior previously reported for DAAO from yeast (Caldinelli et al., 2004). The rates of inactivation at each temperature were determined assaying the enzyme activity up to 20 h. The Arrhenius plot reporting the logarithm of rates of inactivation (ln k) plotted against inverse temperature (1/T) gives a straight line in the 25–55°C range (inset of Figure 2B) pointing to the absence of temperature-induced conformational transitions. From this plot, an activation energy of 119.4 kJ/mol was calculated.

### Modulation of hDAAO activity

**Figure 3:**
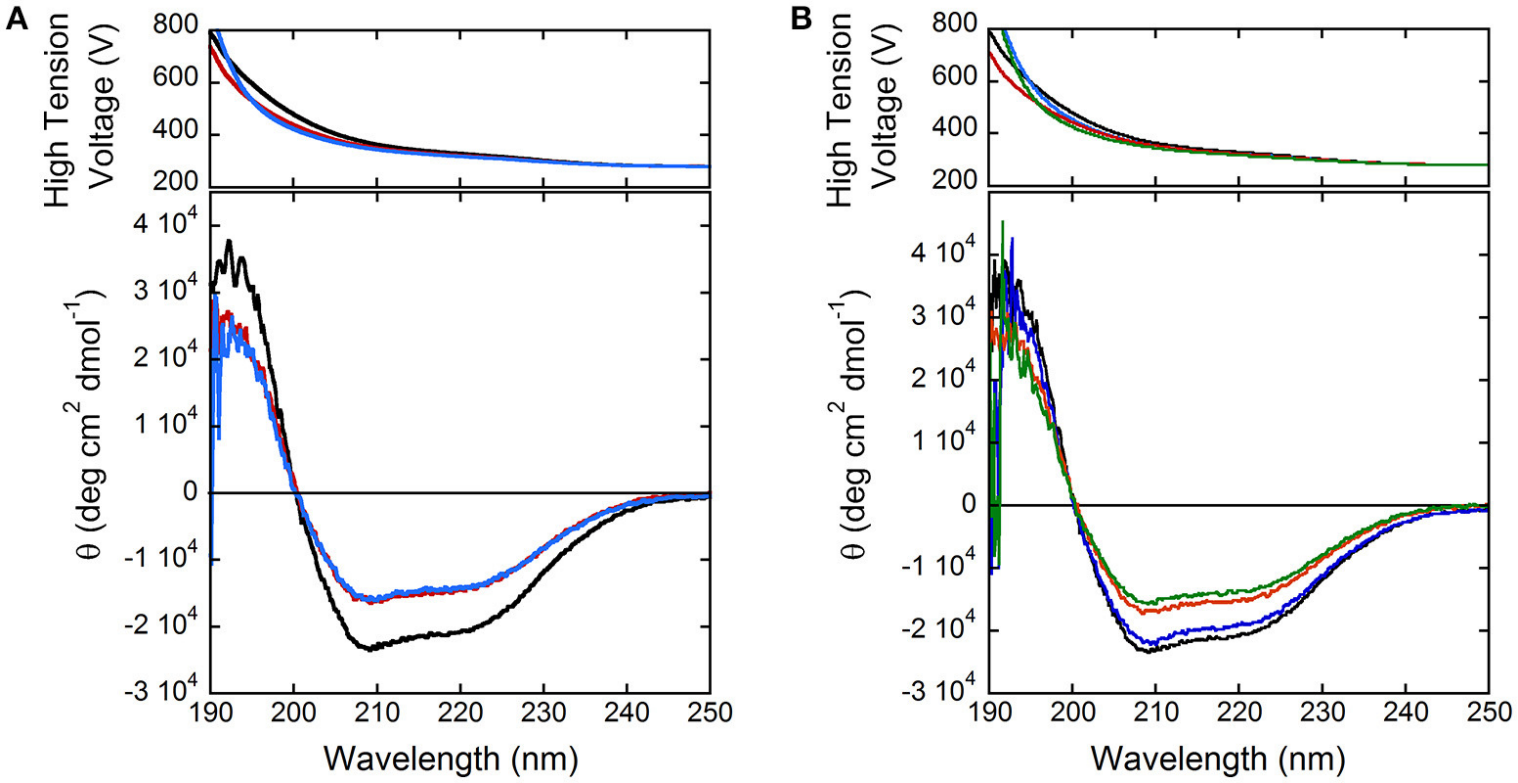
Comparison of far-UV CD spectra of hDAAO holoenzyme (0.1 mg/mL, black) in the presence of: **(A)** 1 and 10 mM calcium ions (red and blue, respectively); **(B)** of 10 mM magnesium ions (blue), 10 μM ATP (red), and of both compounds (green).

In order to shed light on putative regulation mechanisms of hDAAO, the effect of selected physiologically relevant compounds on the flavoenzyme activity was evaluated. The activity and the far-UV CD spectrum of hDAAO are slightly modified by calcium ions at 1 or 10 mM final concentration (Figure 3A), while magnesium ions do not modify both the protein conformation and the hDAAO activity. Adding 10 μM ATP or GTP alter the far-UV CD spectrum of hDAAO, which is similarly affected by the simultaneous presence of 10 μM nucleotide triphosphate and 10 mM magnesium ions (Figure 3B). These ligands do not alter the far-UV CD spectrum of the flavoenzyme (not shown). Notably, we previously demonstrated that pLG72, the specific hDAAO modulator (Sacchi et al., 2008), also interacts with nucleotide triphosphates although no ATPase/GTPase activity was assayed (Sacchi et al., 2017). Indeed, hDAAO activity on D-alanine (at 2 or 28 mM final concentration) is not modified by the simultaneous presence of 10 mM magnesium ions and ATP or GTP (10 μM).

The reducing agent N-acetyl-cysteine (L-NAC, a derivative of the amino acid L-cysteine, an antioxidant and anti-inflammatory agent, which has the ability to modulate NMDAR activity) (Kumar, 2015) at 1 or 10 mM concentration, does not affect hDAAO activity even when added to the enzyme preparation 60 min before the assay.

### Flavin binding

We previously set up a simple procedure to prepare the apoprotein form of hDAAO by dialysis in the presence of 1 M KBr (~70% yield) (Molla et al., 2006). From activity assays and protein fluorescence changes, a K_d_-value of 8 μM for the FAD-apoprotein complex was estimated, which is in good agreement with the value reported by Raibekas et al. (2000). Flavin interaction was significantly strengthened by benzoate binding (K_d_ = 0.3 μM) (Molla et al., 2006; Caldinelli et al., 2010). Here, we decided to investigate the effect of the active-site ligand benzoate on hDAAO flavin binding by following the protein fluorescence quenching during titration of apoprotein with HPLC-purified FAD.

**Figure 4:**
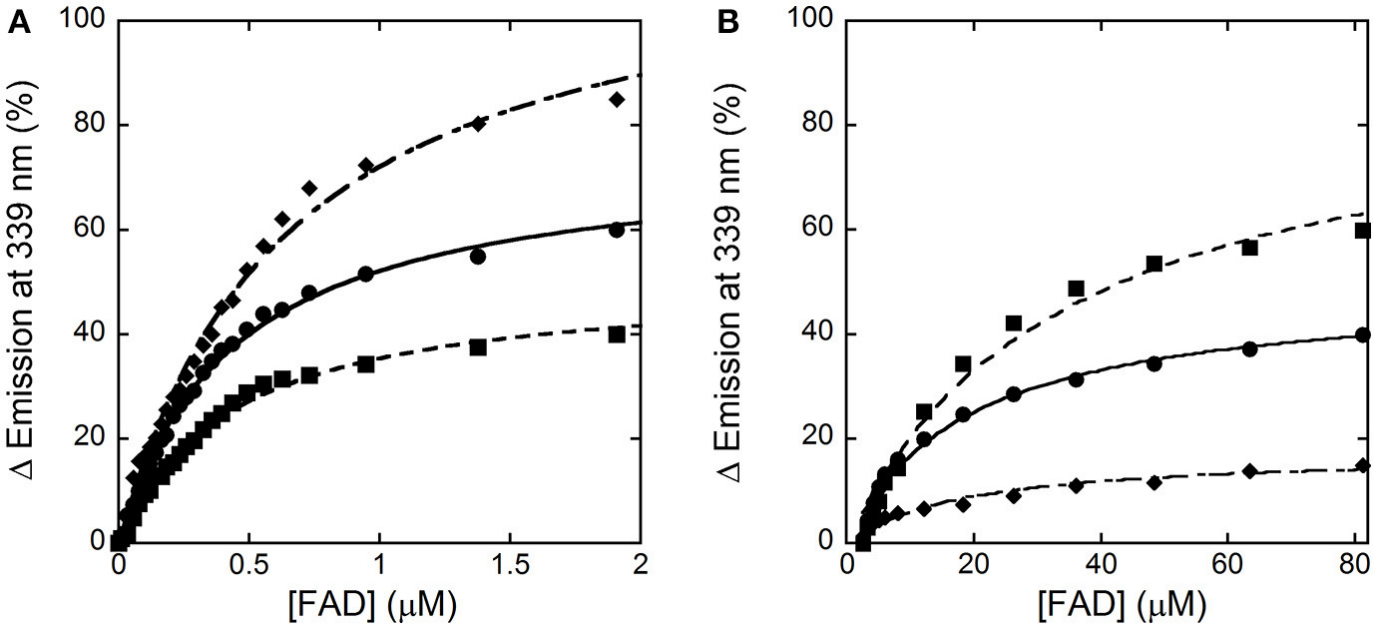
Analysis of FAD binding to hDAAO apoprotein in the absence (■) and presence of 7 μM (●) or 70 μM (♦) of benzoate assessed as protein fluorescence quenching. **(A)** Shows the fit of the values, expressed as percentage of the total change, corresponding to the first saturation phase of the protein fluorescence change (up to 2 μM of cofactor concentration). The curves reported in **(B)** correspond to the best fit for the second phase of saturation. Measurements were performed at 15°C.

In the absence of an active-site ligand, the quenching in protein fluorescence intensity vs. FAD concentration is biphasic: a saturation is apparent up to ~2 μM FAD, corresponding to ~50% of the change in fluorescence intensity, and a second saturation is evident up to ~80 μM FAD (Figure 4). The corresponding dissociation constants were 0.43 ± 0.02 μM and > 30 μM, respectively. Using an amount of benzoate corresponding to the K_d_-value for its binding to hDAAO (7 μM), the change in fluorescence intensity was again biphasic but the first phase accounted for >70% of the total change. K_d_-values of 0.43 ± 0.02 μM and 19.1 ± 2.8 μM were calculated for the first and the second phase, respectively. Notably, using an amount of benzoate corresponding to 10-fold the K_d_ for its binding to hDAAO (i.e., 70 μM), the intensity change in fluorescence was mainly fitted using a single saturation equation with a K_d_-value of 0.48 ± 0.02 μM (Figure 4). The observed increase in the intensity amplitude associated to the first phase indicates that benzoate binding favors a protein conformation with a higher affinity for the cofactor.

The holoenzyme reconstitution process was also investigated by adding increasing concentrations of apoprotein to ~1 μM of FAD and following the decrease in flavin fluorescence at 528 nm (excitation at 450 nm). In the absence of benzoate, the fluorescence change at increasing apoprotein concentration was still biphasic and yielded a K_d, 1_ = 0.67 ± 0.07 μM (corresponding to ~40% of the total change) and a K_d, 2_ = 4.0 ± 0.9 μM. In the presence of 70 μM benzoate the fluorescence intensity change was essentially monophasic: K_d_-value was estimated as 0.40 ± 0.04 μM.

Taken together, these analyses show that the apoprotein form of hDAAO exists in solution as an equilibrium of two conformations differing in FAD binding affinity: an active-site ligand such as benzoate favors the form at higher affinity.

### Substrate specificity

All the known DAAOs show a broad substrate specificity with significant differences depending on the source (Pollegioni et al., 2007; Sacchi et al., 2012). Here, we reviewed published data and accomplished the range of investigated D-amino acids. hDAAO is mainly active on neutral, hydrophobic and slightly polar D-amino acids: the highest catalytic efficiency was apparent with D-cysteine while the same parameter for the brain physiological substrate D-serine was 44-fold lower (see Table 1). The preference of hDAAO for D-cysteine is of utmost relevance (see Discussion). Glycine and D-serine are alternative coagonists that bind to the strychnine-insensitive modulatory site of NMDAR (Mothet et al., 2000). hDAAO is also able to oxidize glycine but the apparent K_m_ for this small substrate (~1,000-fold higher than its physiological concentration) hinders its real degradation by the flavooxidase, especially under physiological conditions.

**Table 1:** Analysis of the substrate specificity of human DAAO.

	**k_cat_ (s^−1^)**	**K_m_ (mM)**	**k_cat_/K_m_ (mM^−1^ x s^−1^)**
**SMALL-MEDIUM SIZE AMINO ACIDS**			
D-Alanine^a^	5.2 ± 0.1 [10]^c^	1.3 ± 0.2 [1]^c^	4.0
D-Serine^a^	3.0 ± 0.1 [4]^d^	7.5 ± 0.5 [1]^c^ [3.9]^d^	0.4
Glycine^a^	~ 0.9	~ 180	0.005
D-Cysteine	8.6 ± 0.2	0.6 ± 0.1	14.6
D-Proline^a^	10.2 ± 0.1	8.5 ± 1.0	1.2
D-Cycloserine	0.77 ± 0.05	83.5 ± 15.0	0.009
**HYDROPHOBIC AMINO ACIDS**			
D-Tyrosine^d^	14.8	1.1	13.5
D-DOPA	40.5 ± 5.1 [21.7]^d^	6.0 ± 1.3 (K_I_ = 41.3) [1.5 (K_I_ = 0.5)]^d^	6.75 [14.4]^d^
D-Phenylalanine^b^	6.6 ± 0.1 [15.5]^d^	2.7 ± 0.2 [1.2]^d^	2.4
D-Tryptophan^b^	3.2 ± 0.1	1.5 ± 0.1	2.1
D-Kynurenine	0.09 ± 0.01	0.7 ± 0.4	0.14
**ACIDIC AMINO ACIDS**			
D-Aspartate^a^	6.7± 1.2	~ 2000	0.0035
D-Glutamate	b. d.	b. d.	
N-Methyl-D-aspartate	b. d.	b. d.	

a *Molla et al., 2006*.

b *Frattini et al., 2011*.

c *Raibekas et al., 2000*.

d *Kawazoe et al., 2007b (at pH 8.3)*.

*b.d., below detection*.

*The apparent kinetic parameters were determined by the oxygen-consumption assay at 21% oxygen saturation, pH 8.5, and at 25°C*.

hDAAO shows a preference for aromatic D-amino acids: the highest maximal activity was determined for D-tyrosine and was high for D-phenylalanine and D-tryptophan as well (Table 1). Notably, hDAAO oxidizes D-kynurenine, i.e., the D-enantiomer of the intermediate found in L-tryptophan degradation pathway: the apparent K_m_-value (0.7 mM) resembles the one determined for D-cysteine. The product of hDAAO-induced D-kynurenine oxidation is kynurenic acid, which is known to inhibit NMDAR by binding to the modulatory glycine site. hDAAO also oxidizes D-cycloserine, an amino acid that acts as NMDAR modulator (Kumar, 2015). A specific argument must be made for the D-enantiomer of 3,4-dihydroxyphenylalanine (DOPA), which can be converted into the L-enantiomer (then used to synthesize dopamine) by the reactions of DAAO and a transaminase. As shown by (Kawazoe et al., 2007b), the apparent maximal activity, as well as the catalytic efficiency k_cat_/K_m_ ratio, is the greatest among tested D-amino acids (Table 1) but the oxidation of this compound is hampered by the observed substrate inhibition effect (K_I_ = 0.5 mM). For sake of comparison, we performed the kinetics on D-DOPA preparations whose purity was evaluated by chiral HPLC: the k_cat_-value for D-DOPA is the highest among the tested compounds and the K_m_ is ~6 mM, thus resulting in a lower catalytic efficiency than that of D-tyrosine. A main difference with previous results concerns the substrate inhibition effect that was observed at a significantly higher D-DOPA concentration (K_i_ = 41.3 mM, Table 1). No difference in kinetic parameters was apparent when the D,L-racemic mixture was used (not shown), suggesting that L-DOPA did not compete with the D-enantiomer for hDAAO binding.

The acidic D-amino acids NMDA and glutamate are not oxidized by hDAAO (at 100 and 500 mM final concentration) while D-aspartate is a very poor substrate: although the maximal activity is quite high (6.7 s^−1^), the catalytic efficiency is strongly affected by the high apparent K_m_ (in the molar range, see Table 1). At physiological concentrations (0.3–0.5 mM) (Weatherly et al., 2017) the activity of hDAAO on D-Asp is negligible.

**Figure 5:**
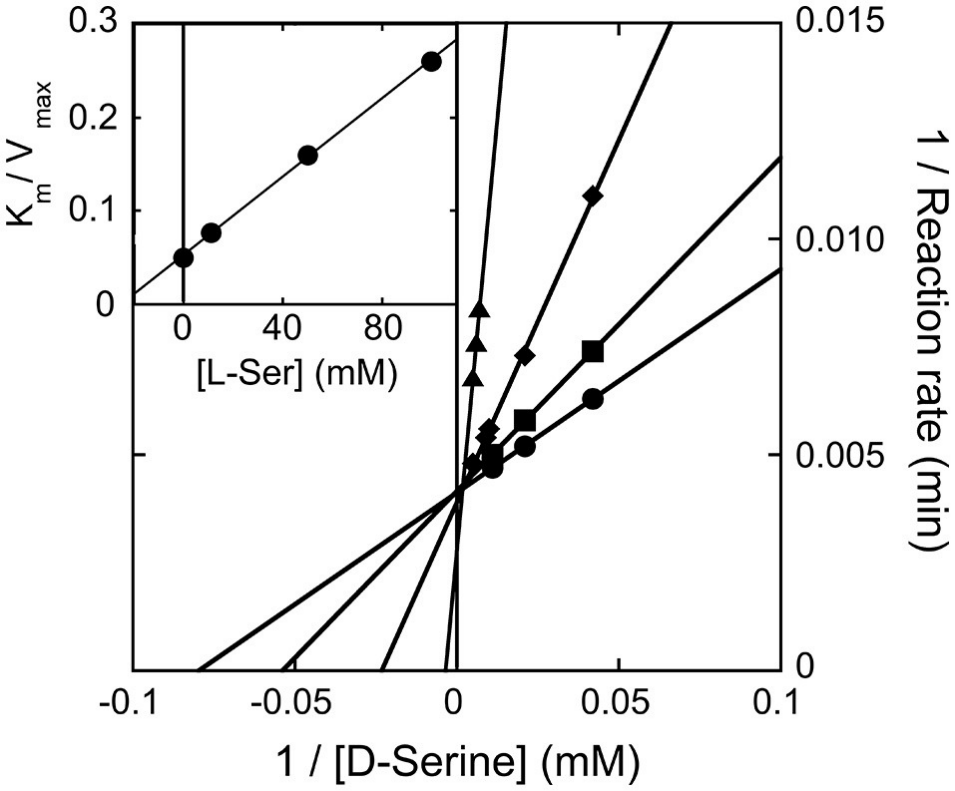
Double reciprocal plot of kinetics of D-serine oxidation by hDAAO in the presence of increasing concentrations of L-serine. K_i_-value for L-serine was estimated by fitting the K_m_/V_max_ values calculated at different L-serine concentrations (see inset).

We observed that the activity of hDAAO on 100 mM D-serine (the physiological substrate in brain) is partially inhibited by the presence of the L-isomer at concentrations higher than 10 mM (not shown): half of the activity measured on D-serine only was assayed when ~500 mM L-serine was simultaneously present. The kinetics of D-serine oxidation by hDAAO was thus investigated at increasing L-serine concentrations: as shown by the double reciprocal plot reported in Figure 5, the L-isomer does not alter the apparent maximal rate but does increase the apparent K_m_-value. Thus, L-serine acts as competitive inhibitor: a K_I_ of 26.2 mM was estimated (see the inset in Figure 5). At physiological concentrations (≤ 2 mM in brain tissues and in blood) (Weatherly et al., 2017), L-serine should not affect the activity of the flavoenzyme on D-serine.

The ability of L-serine to bind at the hDAAO active site like the corresponding D-enantiomer is further confirmed by the observed reduction of FAD following anaerobic addition of the L-amino acid: 14 μM of hDAAO was reduced by 1 mM L-serine in >60 min. A similar behavior was apparent when employing L-alanine or L-valine (not shown) but not NMDA, which is not a substrate of hDAAO.

### Ligand binding

In past years, we focused on the ability of hDAAO to bind small-size molecules (Molla et al., 2006; Caldinelli et al., 2010). The drug chlorpromazine was demonstrated to compete with FAD for the binding to hDAAO apoprotein (Iwana et al., 2008; Sacchi et al., 2008): its binding generated a protein conformation more sensitive to thermal unfolding and proteolysis than the native holoenzyme (Caldinelli et al., 2010). Indeed, a plethora of investigations focused on identifying and characterizing substrate competitive inhibitors, in particular for their potential use as drug to treat schizophrenia and neuropathic pain (Sacchi et al., 2013). By structural bioinformatics, high-throughput screening, and three-dimensional structural studies, a number of DAAO inhibitors have been identified. These compounds have been classified into three distinct chemotypes (Lange et al., 2011): (i) hetero(bi)cyclic carboxylic acids, as 5-methylpyrazole-3-carboxylic acid, 4H-furo[3,2-b]pyrrole-5-carboxylic acid, and 4H-thieno[3,2-b]pyrrole-5-carboxylic acid; (ii) compounds based on benzo[d]isoxazol-3-ol, such as 6-chlorobenzo[d]isoxazol-3-ol (CBIO); and (iii) 3-hydroxyquinoline-2-(1H)-one (a strong hDAAO inhibitor, IC_50_ = 4 nM) and its analogs. For a review focusing on properties and pharmacokinetics of DAAO's inhibitors see (Sacchi et al., 2013).

**Figure 6:**
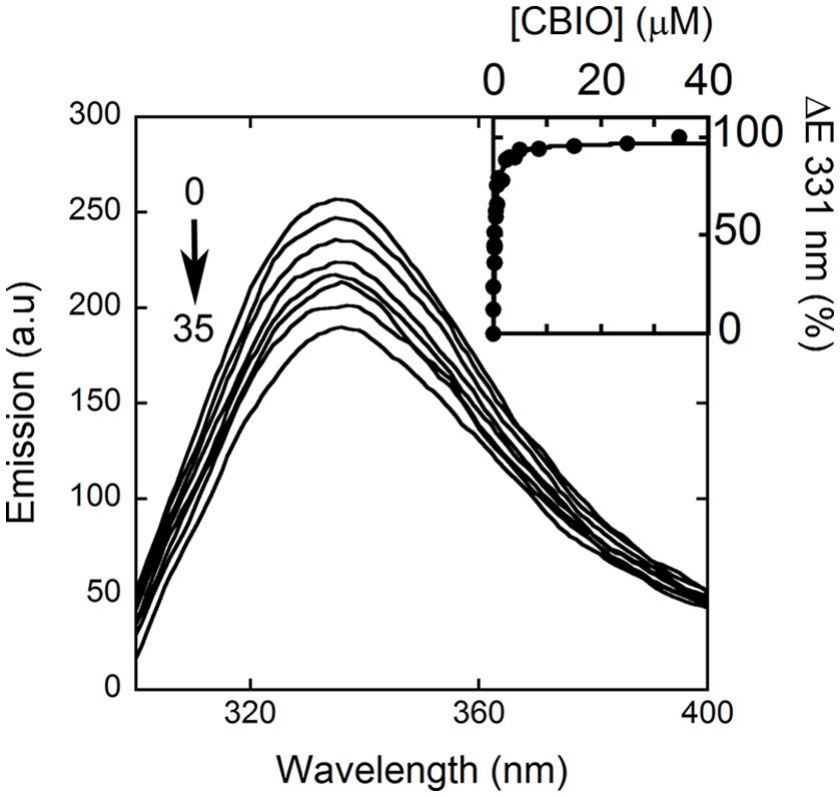
Effect of CBIO binding to the fluorescence emission spectrum of hDAAO after adding increasing concentrations of ligand (up to 35 μM, see arrow). The inset shows the fitting of the fluorescence intensity change at 331 nm (expressed as percentage of maximal change) vs. CBIO concentration. Measurements were performed at 15°C.

**Figure 7:**
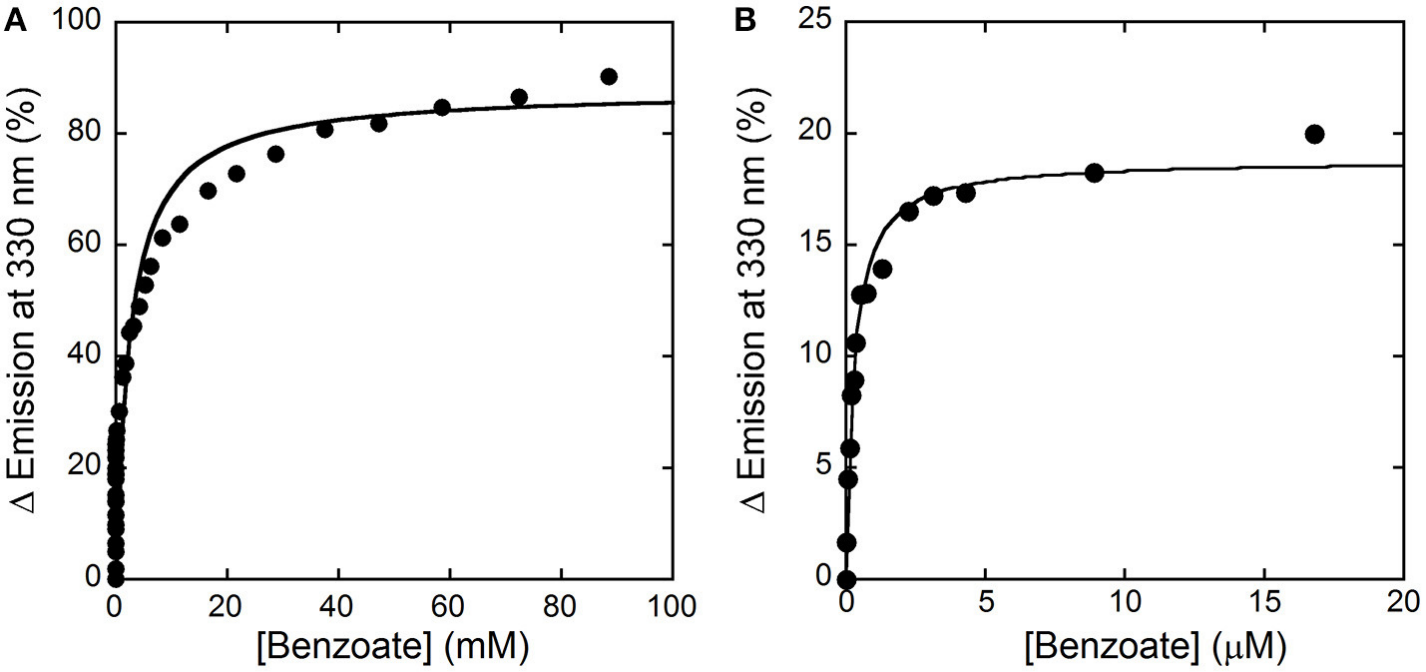
Analysis of benzoate binding to hDAAO holoenzyme. **(A)** Change in protein fluorescence intensity at increasing benzoate concentrations (expressed as percentage of maximal change). **(B)**. Detail of the first saturation phase of the protein fluorescence quenching at a ligand concentration up to ~20 μM. Measurements were performed at 15°C in 20 mM TrisHCl buffer, pH 8.0, 150 mM NaCl, 5% (v/v) glycerol and 5 mM 2-mercaptoethanol.

Here, we decided to investigate the ability of selected compounds to alter hDAAO conformation by following the quenching of protein fluorescence. A decrease in protein fluorescence is apparent at increasing CBIO concentrations: a K_d_ of 0.24 ± 0.05 μM was calculated (Figure 6, inset), suggesting that the induced conformational changes might result in the loss of function. This value is in good agreement with the IC_50_ = 188 nM determined from enzyme's inhibition assays (Ferraris et al., 2008). Similarly, benzoate also decreased the protein fluorescence intensity at ~320–340 nm but a biphasic change was apparent: a first saturation is observed using low benzoate concentrations (≤ 20 μM, K_d_ = 0.33 ± 0.04 μM) and a second saturation, associated to ~80% of fluorescence change, was observed at higher ligand concentrations (K_d_ = 2.59 ± 0.68 mM), see Figure 7. For benzoate binding, a K_d_-value of 5–7 μM was determined following the formation of a shoulder at 484 nm in the absorbance spectrum (Raibekas et al., 2000; Molla et al., 2006), a value of 9.5 μM following the CD signal at ~270 nm (Caldinelli et al., 2010), and a K_I_ of 7 μM following enzyme inactivation (Kawazoe et al., 2006). On the other hand, no change in protein fluorescence was observed with glycine (up to 50 mM), ATP (up to 20 mM), NMDA (up to 50 mM), or D-glutamate (up to 50 mM).

## Discussion

In this work, we biochemically characterized hDAAO in greater depth. Because of its relevant physiological role in the brain related to modulation of the concentration of the NMDAR coagonist D-serine, we were interested to know how hDAAO activity is modulated, taking into consideration both physical-chemical effects and ligands.

The pH and temperature dependence of hDAAO activity and stability was established. The human flavoenzyme shows a good activity on both D-alanine and D-serine in the 6–10 pH range, as well as a good stability (Figure 1). hDAAO is fully stable up to 45°C, a value corresponding to the optimum for the enzymatic activity (Figure 2). Altogether, changes in pH and temperature in the physiological range do not seem a way to modulate the function of this enzyme.

We demonstrated that the flavoenzyme activity is not altered by calcium or magnesium divalent ions and/or nucleotides as well as by the reducing agent L-NAC. In aged mice, the expression of serine racemase (the enzyme responsible for D-serine synthesis) is reduced, whereas no change in DAAO activity was observed (Sato et al., 1996): the chronic treatment of senescent rats with L-NAC to prevent oxidative damage preserved D-serine levels and serine racemase expression, yielding to intact NMDAR activation (Haxaire et al., 2012).

We clarified the substrate acceptance of hDAAO, showing its preference for hydrophobic amino acids: some of them are molecules relevant in neurotransmission as they are intermediates of dopamine biosynthetic pathways (Kawazoe et al., 2007a,b) (see Table 1). Notably, the best substrate is D-cysteine: it was proposed to be converted by DAAO in brain and kidney to 3-mercaptopyruvate, which then generates pyruvate and H_2_S by 3-mercaptopyruvate sulfurtransferase (Shibuya et al., 2013). H_2_S plays different, main physiological roles in neuromodulation, vascular tone regulation, cytoprotection against oxidative stress, angiogenesis, and anti-inflammation; (for a review, see Kimura, 2015). D-Cysteine, originating from food (Friedman, 2010), could thus represent the physiological substrate of hDAAO in specific tissues.

D-Cycloserine is a partial agonist and positive modulator of NMDAR, which binds to the strychnine-sensitive glycine-binding modulatory site: it acts as an antagonist at high doses and as an agonist at low doses (Kumar, 2015). It is oxidized by hDAAO but with a low efficiency because of a very high K_m_-value. Concerning NMDAR modulators, hDAAO is also able to oxidize both glycine and D-aspartate, but again with a very low kinetic efficiency (at least 100-fold lower than the efficiency of D-serine oxidation).

DOPA is a further molecule of relevance for neurotransmission. We confirmed that D-DOPA is oxidized by hDAAO with the highest k_cat_-value (~40 s^−1^): the K_m_ we determined is 4-fold higher than the value previously reported (Kawazoe et al., 2007b). Most interestingly, we demonstrated that D-DOPA is less efficient in enzyme inhibition than previously reported (K_i_ is 41.3 mM) and that L-DOPA does not compete with the D-form for hDAAO active-site binding. In contrast, some L-amino acids can bind in the hDAAO active site, thus acting both as competitive inhibitors (K_I_ for L-serine is 26.2 mM) and, with a very low efficiency, as substrates (by slowly reducing the flavin cofactor under anaerobic conditions).

Concerning the binding of hDAAO inhibitors, a different situation is apparent for benzoate vs. CBIO. While for the latter compound the K_d_-value determined following the quenching of protein fluorescence agrees with the K_d_, IC_50_, and K_i_-values reported in the literature, a process characterized by two phases is apparent for benzoate binding. This observation indicates that two protein conformations in equilibrium with different affinity for the ligand or that two ligand binding sites exist. We demonstrated that benzoate interacts with both the holo- and the apoprotein forms of hDAAO (Caldinelli et al., 2009). Noteworthy, a recent investigation combining *in silico* docking simulation and labeling experiments using 4-bromo-3-nitrobenzoic acid highlighted the presence of a second ligand binding site, located in a cleft between monomers (Kohiki et al., 2017).

Concerning flavin binding, we previously demonstrated that an active-site ligand stabilizes the apoprotein-FAD interaction (Molla et al., 2006; Caldinelli et al., 2010) pushing the holoenzyme formation. Here, we clearly demonstrated that, in solution, hDAAO apoprotein exists in two alternative conformations with different flavin affinity: the equilibrium is shifted toward the one at higher avidity by the presence of the competitive inhibitor benzoate, reaching a K_d_-value similar to that of rat or pig DAAOs (Frattini et al., 2011). It is noteworthy that the reconstitution process leading to the active holoenzyme is a biphasic, sequential process; first, the apoprotein binds FAD and gains catalytic activity—and this step is 20-fold faster when benzoate is bound (Caldinelli et al., 2010)—and then a slower secondary conformational change follows, which stabilizes the protein (Caldinelli et al., 2009). Although no crystal structure of hDAAO in the absence of an active-site ligand has been solved, our biochemical evidence suggests that, in addition to the known conformation of the hydrophobic region ^47^VAAGL^51^ at the *si*-face of the isoalloxazine ring of FAD observed in the hDAAO-benzoate complex, a second conformation should exist that possesses a lower affinity for the flavin cofactor. This is an important issue since the holoenzyme stability both increases the half-life of the protein and affects the cellular D-serine level (Caldinelli et al., 2010).

Taken together, these findings strengthen our belief that, in the case of hDAAO, evolution adopted sophisticated regulatory strategies. Because of the weak interaction between FAD and hDAAO apoprotein and of the physiological concentration of the cofactor in human brain tissues, the flavoenzyme is largely present in the apoprotein, inactive form. The flavin binding and the kinetic properties (hDAAO possesses a low catalytic efficiency and substrate affinity) seem to be evolved to generate an enzyme that controls D-serine concentration in brain, avoiding an excessive degradation of the neuromodulator. The fact that the presence of a ligand in the active site promotes a conformational switch toward a protein form able to bind more avidly the cofactor might represent a mechanism finely modulating hDAAO activity: the free enzyme, significantly present in solution as inactive apoprotein, is rapidly turned into the flavinylated, active holoenzyme by the presence of the substrate. This, in turn, would represent an effective way to keep the concentration of specific D-amino acids in distinct tissues in the physiological range. In conclusion, hDAAO possesses a number of peculiar properties that distinguish it from homologs from diverse sources. Such properties make this flavoenzyme an interesting protein that can be used by different tissues to meet various physiological needs related to the catabolism of different D-amino acids.

## Author contributions

GM performed the experiments, analyzed the data and wrote the paper; MV purified the recombinant enzyme; SS designed the study and analyzed the data; LP designed the study, analyzed the data and wrote the paper.

### Conflict of interest statement

The authors declare that the research was conducted in the absence of any commercial or financial relationships that could be construed as a potential conflict of interest.

## Abbreviations

CBIO: 6-chloro-benzo(d)isoxazol-3-ol
CD: circular dichroism
DAAO: D-amino acid oxidase (EC 1.4.3.3)
DOPA, 3: 4-dihydroxyphenylalanine
hDAAO: human D-amino acid oxidase
L-NAC: N-acetyl-L-cysteine
NMDAR: N-methyl-D-aspartate subtype of glutamate receptor.
